# Flavocoxid, a Nutraceutical Approach to Blunt Inflammatory Conditions

**DOI:** 10.1155/2014/790851

**Published:** 2014-08-24

**Authors:** Alessandra Bitto, Francesco Squadrito, Natasha Irrera, Gabriele Pizzino, Giovanni Pallio, Anna Mecchio, Federica Galfo, Domenica Altavilla

**Affiliations:** ^1^Department of Clinical and Experimental Medicine, Section of Pharmacology, University of Messina, Torre Biologica 5th Floor, AOU Policlinico “G. Martino”, Via C. Valeria Gazzi, 98125 Messina, Italy; ^2^Department of Paediatric, Gynaecological, Microbiological and Biomedical Sciences, University of Messina, Via C. Valeria Gazzi, 98125 Messina, Italy

## Abstract

Flavonoids, from *Scutellaria baicalensis* (Chinese skullcap) and *Acacia catechu* (black catechu), have been shown to exert a variety of therapeutic effects, including anti-inflammatory, antiviral, antibacterial, and anticancer activities. Flavocoxid is a mixed extract containing baicalin and catechin and it acts as a dual balanced inhibitor of cyclooxygenase-1 (COX-1) and COX-2 peroxidase enzyme activities with a significant inhibition of 5-lipoxygenase (5-LOX) enzyme activity *in vitro*. Flavocoxid downregulates gene or protein expression of several inflammatory markers and exerts also strong antioxidant activity in several experimental models. Controlled clinical trials and a postmarketing study have clearly shown that flavocoxid is as effective as naproxen in managing the signs and symptoms of osteoarthritis of the knee and it has better upper gastrointestinal, renal, and respiratory safety profile than naproxen. Flavocoxid may therefore provide a potential therapeutic approach to the treatment of chronic inflammatory conditions.

## 1. Introduction

**Figure 1:**
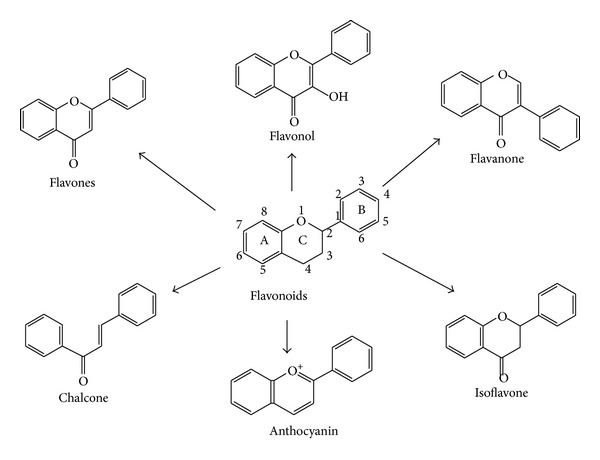
Basic chemical structures of different natural occurring flavonoids.

**Figure 2:**
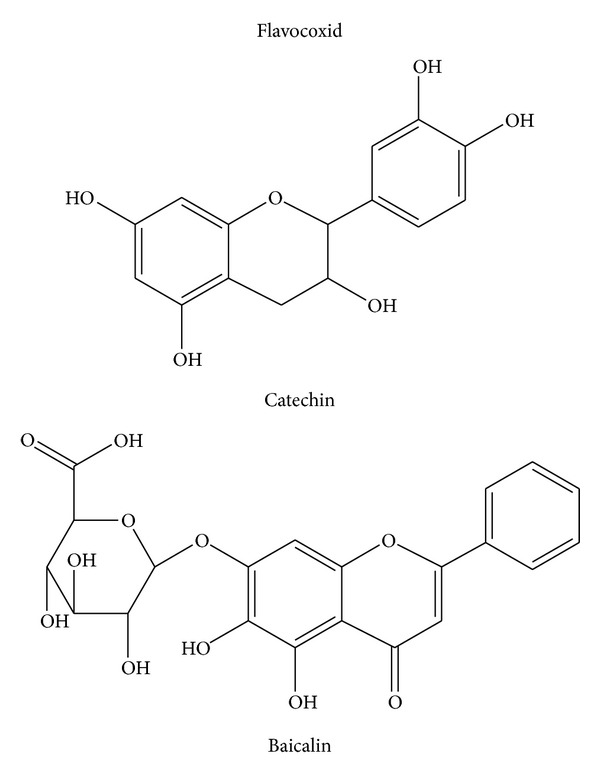
Flavocoxid components: catechin and baicalin.

Inflammation is considered the main response of the body evoked to deal with injuries and its hallmarks include swelling, redness, pain, and fever [1]. Several mediators regulate the events of acute inflammation, influence vascular changes, and provoke inflammatory cell recruitment [1–3]. The inflammatory response is a complex self-limiting process precisely regulated to prevent extensive damage of the host. When the self-limiting nature of this protective mechanism is inappropriately regulated, it is transformed to a detrimental chronic state of inflammation. Several diseases are associated with chronic inflammation, including osteoarthritis, atherosclerosis, diabetes, obesity, Crohn's disease, and cancer [1–3]. Although the today used steroidal anti-inflammatory drugs (SAID) and nonsteroidal anti-inflammatory drugs (NSAIDs) effectively manage the acute inflammatory reaction, in chronic inflammatory states the long-term treatment is followed by severe adverse effects. This justifies the search for innovative and safe anti-inflammatory agents. Attractive research candidates are plant constituents. Recently, there has been interest in the potential of flavonoids to modulate the chronic inflammatory reaction. Flavonoids are major constituents of fruits, vegetables, and beverages, such as wine, tea, cocoa, and fruit juices. Flavonoids have a similar structure consisting of two aromatic rings (A and B), which are bound together by three carbon atoms, forming an oxygenated heterocycle (ring C; Figure 1). According to changes in the chemical structure, flavonoids are divided into seven classes: flavonols, flavones, flavanones, flavanonols, flavanols, anthocyanidins, and isoflavones [4]. The flavanols, also referred to as flavan-3-ols, are mainly present in green and black tea, red wine, chocolate, and fruits such as apples, grapes, and strawberries. Typical dietary flavanols include catechin, epicatechin, epigallocatechin (EGC), and epigallocatechin gallate (EGCG). Flavones, such as apigenin and luteolin, are found in parsley, chives, artichoke, and celery. Dietary flavanones include naringenin, hesperetin, and taxifolin and are found mainly in citrus fruit and tomatoes. Finally, isoflavones such as genistein and daidzein are a subclass of the flavonoids family found in soy and soy products. They have a large structural variability and more than 600 isoflavones have been identified to date and are classified according to oxidation level of the central pyran ring (Figure 1).* Acacia catechu*, also called black catechu, is a traditional medicinal plant commonly used in Indian subcontinent and Southeast Asia, with antipyretic, antidiarrheal, hypoglycaemic, and hepatoprotective activities [5, 6]. The major chemical components in an* Acacia catechu* extract are catechin and epicatechin-3-O-gallate. Catechin reduces inflammation and blunts the production of proinflammatory cytokines, such as interleukin-1 (IL-1) and tumor necrosis factor-*α* (TNF-*α*), as demonstrated in rats with experimental arthritis [7].* Scutellaria baicalensis*, a traditional Chinese herbal medicine, also called Chinese skullcap, has been used to treat respiratory inflammatory diseases [8–12], viral infections [13–15], cardiovascular diseases [16–18], and cancer [19, 20]. Baicalin, extracted by* Scutellaria baicalensis*, inhibits cyclooxygenase COX-1 and COX-2 peroxidase and 5-lipoxygenase (5-LOX) enzyme activities, decreases production of proinflammatory eicosanoids, and attenuates edema in an* in vivo* model of inflammation [21]. The anti-inflammatory activity of baicalin was also associated with a binding inhibition of chemokines, such as macrophage inflammatory protein (MIP)-1*β*, monocyte chemotactic protein-2 (MCP-2), causing a reduced capacity of the chemokines to induce cell migration [21]. The flavonoids, baicalin and catechin, modulate the activities of arachidonic acid, metabolizing enzymes involved in this pathway, and iNOS (NO producing enzyme). Flavocoxid is a mixture of the flavonoid molecules catechin, from* Acacia catechu*, and baicalin, extracted from* Scutellaria baicalensis*, concentrated to greater than 90% purity (Figure 2). Thanks to its components flavocoxid, in addition to anti-inflammatory properties, may also act as an antioxidant, reducing reactive oxygen species including hydroxyl radical, superoxide anion radical, and hydrogen peroxide. The combined effect of the 2 flavonoids is greater than the isolated molecules, as shown in a recent paper [22]. As a consequence of NF-*κ*B modulation, flavocoxid reduces COX-2, 5-LOX, iNOS, and TNF*α* production, and it also blunts the formation of COX-2, 5-LOX, and iNOS metabolites, as PGE2, LTB4, and nitrates [22–24]. These effects provide a rationale for the use of a dual inhibitor in acute and chronic inflammatory conditions. Flavocoxid, marketed as Limbrel in the USA, is a USFDA-regulated prescription, and it is expected to have significant therapeutic efficacy in the managing of chronic inflammation. This review will focus on the preclinical pharmacology, toxicology, and clinical pharmacology of this new attractive compound.

## 2. Preparation of Flavocoxid

Flavocoxid is prepared from roots of* Scutellaria baicalensis* and* Acacia catechu* (US patent number 7,514,469). The roots of* Scutellaria baicalensis* are extracted with 70% ethanol and then recrystallized with an ethanol/water solvent [23]. The* Scutellaria baicalensis* extract contains baicalin as the major component and additional minor free-B-ring flavonoids. In the roots of* Acacia catechu*, (+)-catechin is the major component at a content of 80%. Minor amount of its enantiomer epicatechin and other minor amounts of flavans are present [23]. Analysis of the extracts is carried out separately by high performance liquid chromatography with photodiode array detector (HPLC/PDA) and liquid chromatography-mass spectrometry (LC/MS) and indicates a major compound, baicalin, from* Scutellaria baicalensis* extract and (+)-catechin from* Acacia catechu* extract by comparison with known standards. The presence of these compounds is then confirmed by carbon nuclear magnetic resonance (^13^C-NMR) and proton nuclear magnetic resonance (^1^H-NMR) analysis, respectively. The final flavocoxid formulation (Figure 2) is a mixture of >90% purified baicalin and catechin with the remainder being excipient (5-6%) and water (3%). Confirmation of the combined flavonoids content can be obtained by HPLC analysis. Both flavonoids are detected using UV detector at 275 nm and identified based on retention time by comparison with known flavonoids standards [23]. These ingredients are generally recognized as safe (GRAS). For an ingredient to be recognized as GRAS by the US Food and Drug Administration (FDA), it requires technical demonstration of nontoxicity and safety, general recognition of safety through widespread usage, and agreement of that safety by experts in the field.

## 3. Effects of Flavocoxid on Arachidonic Acid Formation and Metabolism

A series of* in vitro* and* in vivo* experiments have been carried out in order to dissect out the exact mechanism of action of flavocoxid. First of all, flavocoxid was tested in peritoneal macrophages (MΦ) stimulated with lipopolysaccharide (LPS) to investigate a possible effect on phospholipase A_2_ (PLA_2_) activity [24]. Flavocoxid did not significantly modify cell viability at 200 and 500 *μ*g/mL, but it markedly inhibited PLA_2_ activity (IC_50_ = 60 *μ*g/mL) at doses of 50, 100, 200, and 500 *μ*g/mL [24]. This finding indicates that flavocoxid modulates the generation of AA from membrane phospholipids caused by tissue damage during chronic inflammation. The effects of flavocoxid on COX-1 and COX-2 enzyme activities were also investigated in dedicated* in vitro* enzyme assays [24]. COX proteins have two different enzymatic moieties for AA metabolism: the cyclooxygenase (CO) one and the peroxidase one (PO). The CO activity converts AA to PGG_2_ and the PO activity transforms PGG into PGH_2_. Finally, cell synthases and isomerases convert PGH_2_ to thromboxanes (TXB), prostaglandins (PG), and prostacyclin (PGI). Experiments were carried out to investigate the specific inhibitor effects of flavocoxid on CO and PO enzyme moieties of either COX-1 or COX-2. The compound had no significant anti-CO COX-2 activity up to 50 *μ*g/mL. In addition, flavocoxid showed CO COX-1 IC_50_ of 25 *μ*g/mL, while indomethacin has a CO COX-1 IC_50_ of 0.012 *μ*g/mL, thus suggesting that flavocoxid has little anti-CO activity on both COX enzymes compared to well-known anti-inflammatory agents. Imbalances in COX-2 versus COX-1 inhibition caused by selective COX-2 lead to production of several AA metabolites responsible for an enhanced risk of edema, hypertension, and myocardial infarction [25]. Flavocoxid produced a balanced inhibition of both COX-1 and COX-2 PO activities, with IC_50_ of 12.3 and 11.3 *μ*g/mL, respectively. This clearly indicates that flavocoxid exerts its activity via modulation of the PO activity of these enzymes. Currently available NSAIDs and COX-2 inhibitors do not influence the 5-LOX pathway and therefore do not block the production of the dangerous leukotrienes (LTs) that cause vasoconstriction and leukocyte attraction and accumulation. Furthermore COX-1 and COX-2 blockade by NSAIDs or selective COX-2 inhibitors causes a shunting of the AA metabolism towards the 5-LOX pathway, thus inducing an overproduction of these fatty acid mediators which can cause multiple organ damage [26]. Enhanced levels of LTs are also measured in a variety of pathological conditions such as asthma, gastric ulceration, renal insufficiency, and cardiovascular complications [27, 28]. Flavocoxid was incubated along with purified 5-LOX enzyme in the presence of an oxygen sensing chromagen* in vitro* to study the formation of unstable hydroperoxyeicosatetraenoic acids (HPETEs) intermediates in the synthesis of LTs. Flavocoxid inhibited the 5-LOX enzyme showing an IC_50_ of 110 *μ*g/mL. Phenidone, a well-known 5-LOX inhibitor used as a positive control in theses assays, had an IC_50_ of 1.3 *μ*g/mL. No other NSAIDs or selective COX-2 inhibitors, including rofecoxib, valdecoxib, diclofenac, meloxicam, and aspirin, displayed an anti-5-LOX activity. All these findings, taken together, indicate that flavocoxid also reduces the production of LTBs from 5-LOX and may avoid the deleterious accumulation of these lipid mediators caused by the NSAIDs-induced 5-LOX shunt. Nonenzymatic lipid peroxidation is another important pathway of the AA metabolism. Reactive oxygen species (ROS) may react with exaggerated AA levels leading to the production of F2-isoprostanes and 4-hydroxynonenal (HNE) together with enhanced malondialdehyde levels. All these markers of oxidative stress are elevated during chronic inflammation and contribute to the pathological cascade leading to organ damage and dysfunction. Therefore, it is relevant for an anti-inflammatory agent to possess an antioxidant effect. Experiments were carried out to analyze this issue, and the* in vitro* antioxidant activity of flavocoxid was evaluated using oxygen radical absorbance capacity (ORAC) procedures and was compared with that of well-known antioxidants such as vitamin C and vitamin E. The ORAC analysis provides a measure of the scavenging capacity of antioxidants against the peroxyl radical. Trolox, a water-soluble vitamin E analog, is used as the calibration standard, and the ORAC result is expressed as *μ*molTE/g dry weight. The ORAC_total_ for flavocoxid (3719 *μ*molTE/g) was significantly greater than that of either vitamin C (2000 *μ*molTE/g) or vitamin E (1100 *μ*molTE/g). Flavocoxid showed also high values for the ferric reducing/antioxidant power (FRAP), the peroxynitrite radical averting capacity (NORAC), and the Trolox equivalent antioxidant capacity (TEAC), thus clearly showing that this compound exerts a strong antioxidant activity [24].

## 4. Effects of Flavocoxid on Proinflammatory Gene Expression

The effects of flavocoxid on gene and protein expression of inflammatory markers were studied in rat peritoneal macrophages stimulated with *Salmonella enteritidis *LPS [29]. In fact it is worthy of interest to identify for an anti-inflammatory agent a mechanism of action at the level of inflammatory gene and protein expression. Peritoneal macrophages had a constitutive expression of COX-1 and LPS did not modify it. By contrast LPS stimulation of peritoneal macrophages resulted in a marked increase in both COX-2 and 5-LOX expression [29]. Flavocoxid attenuated, in a concentration dependent manner, the increase in COX-2 and 5-LOX expression [29]. Together with LPS-induced COX-2 and 5-LOX activation, the generation of PGE_2_ and LTB_4_ was markedly augmented. Flavocoxid significantly reduced the increase in PGE_2_ and LTB_4_. In addition, LPS-primed macrophages had an enhanced mRNA expression for iNOS and augmented nitrate content; flavocoxid significantly blunted, in a concentration dependent manner, the increase in iNOS and nitrate production. These results [22, 24, 29] suggest that flavocoxid inhibits COX-2, 5-LOX, and iNOS gene activation and abrogate the biosynthesis of related inflammatory mediators. Among these mediators an important role is played by TNF-*α*, a pleiotropic proinflammatory cytokine [30], markedly induced in peritoneal macrophages stimulated with LPS. Flavocoxid caused a significant and concentration dependent reduction in the levels of TNF-*α* mRNA and in the formation of the mature protein. Nuclear factor kappa-B (NF-*κ*B) plays a prominent role in the inflammatory cascade [31]; it is an important transcription factor complex that regulates the expression of several genes involved in immune and inflammatory responses during chronic human diseases [32, 33]. In unstimulated cells, NF-*κ*B is constitutively localized in the cytosol as a heterodimer by physical association with an inhibitory protein, called I*κ*B*α*. Following activation, the NF-*κ*B heterodimer is rapidly translocated to the nucleus where it activates the transcription of target genes, including the genes encoding for proinflammatory cytokines, adhesion molecules, chemokines, and inducible enzymes (such as COX-2, 5-LOX, and iNOS). Flavocoxid was shown to reduce I*κ*B*α* loss from the cytoplasm and to blunt NF-*κ*B binding to DNA in LPS-stimulated macrophages [29]. Therefore flavocoxid, acts at gene and protein expression level through NF-*κ*B activity inhibition, blocking the auto-amplifying loop during the inflammatory response.

## 5. Effects of Flavocoxid on Experimental Models of Inflammation

**Figure 3:**
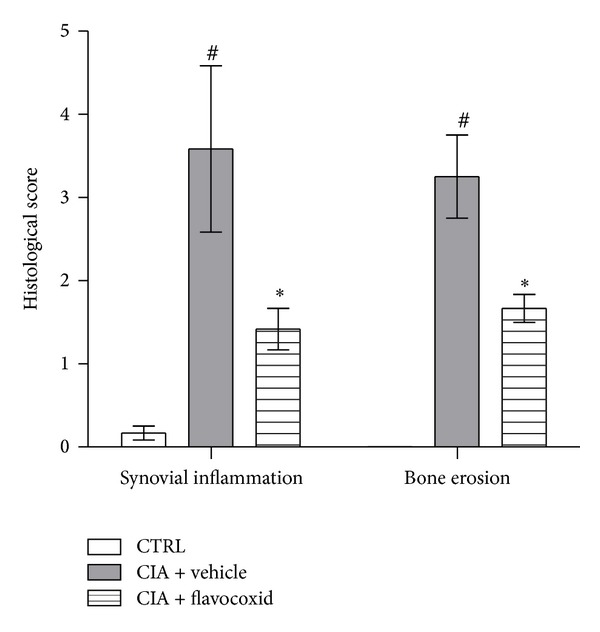
Effects of flavocoxid in a collagen-induced arthritis model. ^#^ *P* < 0.001 versus ctrl; *P < 0.005 versus CIA + vehicle.

Flavocoxid was studied in animal experimental models of inflammation aimed to test its efficacy. In a first experiment, collagen-induced arthritis (CIA) was induced in DBA/1 mice by an intradermal injection of an emulsion containing bovine type II collagen in complete Freund's adjuvant. CIA animals were then randomized to receive vehicle or flavocoxid (20 mg/kg) and the treatment lasted 45 days (Figure 3, data on file). Flavocoxid reduced PGE_2_ and LTB_4_ levels, significantly ameliorated the clinical signs of arthritis, improved the histological damage, decreased the cartilage expression and the circulating levels of several markers of severity disease including TNF-*α*, IL-6, high mobility group box-1 (HMBG-1), and also caused an enhanced expression of the anti-inflammatory cytokine IL-10. Interestingly, flavocoxid positively modulated the balance between receptor activator of nuclear factor-*κ*B ligand (RANKL) and osteoprotegerin (OPG), a cytokine system involved in bone and cartilage remodeling. Collectively these experimental findings demonstrate that flavocoxid is a valuable therapeutic agent in the treatment of inflammatory conditions, including arthritis and osteoarthritis.

The dual inhibitor was also tested in acute inflammatory diseases such as acute pancreatitis, an autodigestive inflammatory disease, that causes acinar cell damage and finally culminates in hemorrhagic necrosis of the pancreas and eventually multiple organ failure [34]. A large body of evidence suggests that upregulation of inflammatory mediators, including COX-2, 5-LOX, cytokines, and chemokines, orchestrates this pathological process [35]. Acute pancreatitis can be induced in rats by injection of cerulein, a secretagogue agent. Flavocoxid was investigated for its effects in cerulein-induced pancreatitis [36] at a dose of 20 mg/kg; it inhibited COX-2 and 5-LOX expression and reduced serum levels of lipase and amylase and the degree of pancreatic edema. Administration of flavocoxid also blunted the increased pancreatic TNF-*α* mRNA expression, serum LTB_4_ and PGE_2_ levels, and protected against histological damage in terms of vacuolization and leukocyte infiltration. These interesting findings may provide a potential therapeutic approach to the treatment of patients at high risk of developing this life-threatening condition.

Flavocoxid was also tested in a degenerative chronic disease, Duchenne muscle dystrophy (DMD), a progressive muscle-wasting disease leading to death, usually in early adulthood [37]. The disease results from absence of the protein dystrophin, which is an essential component of the dystrophin-glycoprotein complex that maintains membrane integrity of muscle fibers by linking cytoskeleton to extracellular matrix. Muscle degeneration in muscle dystrophy is exacerbated by the endogenous inflammatory response and increased oxidative stress, and NF-*κ*B plays a pivotal role in orchestrating this inflammatory cascade [38, 39]. The effects of flavocoxid were studied in a comparison study with methylprednisolone, the gold standard treatment for DMD patients, using the mdx mice, the murine model of DMD [37]. Five-week-old mdx mice were treated for 5 weeks with flavocoxid, methylprednisolone, or vehicle. Flavocoxid was more effective than methylprednisolone in ameliorating functional properties both* in vivo* and* in vitro*; in reducing serum creatine kinase (CK), a marker of muscle necrosis; in blunting the expression of oxidative stress markers and inflammatory mediators. Furthermore more efficiently than methylprednisolone, flavocoxid inhibited NF-*κ*B and mitogen-activated protein kinases (MAPKs) signal pathways, decreased muscle necrosis, and augmented muscle regeneration [37]. This experiment suggests that flavocoxid counteracts the chronic inflammatory cascade that contributes to muscle necrosis and degeneration in DMD showing a degree of activity higher than that of methylprednisolone. Since methylprednisolone possesses also important side effects, flavocoxid with a better safety-efficacy profile might represent a valuable alternative in the treatment of DMD. However this hypothesis deserves to be confirmed in a clinical setting.

In another experimental model of chronic inflammation, benign prostatic hyperplasia (BPH), we tested the effects of flavocoxid since COX and 5-LOX are significantly elevated in the overgrowing prostate [40]. Therefore a “dual inhibitor” of the COX and 5-LOX enzymes might be of benefit in this disease. Rats were treated, daily, with testosterone propionate (3 mg/kg/sc) or its vehicle for 14 days. Testosterone administered animals were randomized to receive vehicle (1 mL/kg, ip) or flavocoxid (20 mg/kg, ip) for 14 days. Flavocoxid reduced prostate weight and hyperplasia, blunted the augmented expression of COX-2 and 5-LOX as well as the increased production of PGE_2_ and LTB_4_, enhanced the proapoptotic Bax and caspase-9, and decreased the antiapoptotic Bcl-2 mRNA. Flavocoxid reduced also the epidermal growth factor (EGF) and vascular endothelial growth factor (VEGF) expression. The data obtained from this study would indicate that a “dual inhibitor” of the COX and 5-LOX enzymes, such as flavocoxid, might represent a rationale therapeutic approach to reduce benign prostate growth [40].

## 6. Toxicity Studies on Flavocoxid

A toxicological testing of the relative pure combination of baicalin and (+)-catechin has been performed* in vitro* and in experimental animals [41, 42]. THP-1 cells, a human immortalized monocyte cell line, were used to assess cytotoxicity in an LDH assay. When cells were grown to confluent monolayer and then exposed to increasing concentration of several NSAIDs, celecoxib, and the baicalin/(+)-catechin combination, only indomethacin and celecoxib showed significant cytotoxicity above 50 *μ*g/mL. Flavocoxid showed low cytotoxicity at the highest test concentration (100 *μ*g/mL) [41]. Acute (2000 mg/kg/day for 14 consecutive days) and subchronic (50, 250, and 500 mg/kg/day) toxicity study in mice revealed no abnormalities in any toxicological end points examined including animal body weight, gross organ pathology and tissue histology, and blood chemistry or serology [42]. Flavocoxid was also administered in Fisher 344 rats, a model for gastric toxicity of NSAIDs, and showed no sign of ulceration [41]. The baicalin/(+)-catechin combination was tested at a 10 *μ*M concentration in a liver microsomal assay with endoplasmic reticulum fractions using spectrophotometric quantization of 7-benzyloxy-4-(trifluoromethyl)-coumarin as the substrate for CYP450 profiling. Flavocoxid showed only moderate inhibition of CYP1A2 (23%) at a 10 *μ*M concentration and low inhibition for all other CYP isoforms (11/16%). The combined extract also showed no mutagenicity in the AMES test [41]. An additional 90-day oral toxicity study was carried out in Hsd : SD rats to determine the potential of the baicalin/(+)-catechin combination to produce gastric toxicity [42]. Flavocoxid was administered at the dose levels of 250, 500, and 1000 mg/kg/day. There were no flavocoxid-related adverse events, including mortality, changes in body weight and food consumption, neurological effects (assessed by the Functional Observational Battery and motor activity), organ weight changes, or histopathological alterations. In addition flavocoxid caused no change in sperm count and comparable estrus aging [42]. This study identified a dose of 1000 mg/kg/day/as the no-observed-adverse-effect level (NOAEL). This promising preclinical safety profile encourages the use of flavocoxid in humans.

## 7. Clinical Evidence Supporting Flavocoxid Efficacy and Safety

A study was designed to compare the effectiveness and safety of flavocoxid to naproxen in subjects with moderate to severe osteoarthritis (OA) of the knee [43, 44]. This was a randomized, multicenter, and double-blind study involving 220 subjects with OA of the knee that were randomized to receive either flavocoxid (500 mg twice daily) or naproxen (500 mg twice daily for 12 weeks). Primary outcome measures included the Western Ontario and McMaster Universities Osteoarthritis index (WOMAC) and subscales and timed walk. More than 90% of the subjects in both groups had significant reduction in the signs and symptoms of OA. No statistical significant difference in efficacy between flavocoxid and naproxen was observed [43, 44]. Flavocoxid treated patients had also significantly fewer upper gastrointestinal, renal, and respiratory adverse events (AEs). These results indicate that flavocoxid is effective as naproxen in the management of OA of the knee and shows a better safety profile than naproxen. An additional study in healthy volunteers found that flavocoxid does not affect the primary or extrinsic pathway of hemostasis and, by not inhibiting the anticoagulation effects of aspirin, may have utility in cardiovascular patients with chronic inflammation [45]. An open-label, postmarketing study (GOAL: Gauging Osteoarthritis [OA] with Limber) was performed to determine the overall efficacy and gastrointestinal tolerability of flavocoxid [46]. A total of 1067 patients at 41 rheumatology practices were enrolled and prescribed flavocoxid 500 mg b.i.d. for 60 days. The Physician Global Assessment of Disease (PGAD) visual analog scale (VAS) was used as a global measure to assess the signs and the symptoms of OA including joint discomfort, functional stiffness, functional mobility, and quality of life. Furthermore both overall tolerability and upper GI tolerability were assessed by individual questions scored on a 5-part Likert scale. Physicians were also asked to monitor any interruption in or cessation of use of flavocoxid due to the GI symptom as well as changes in the use of gastroprotective medications. A close monitoring of adverse events (AEs) was also carried out. In the 1005 patients who completed all the follow-up visits there was a significant improvement in VAS score. The most important improvement was observed in patients with moderate to severe OA and in those subjects that were historically nonresponders to NSAIDs. A low incidence of o AEs was also observed with a good overall and GI tolerability. In addition the use of flavocoxid resulted in a >30% reduction in or cessation of the gastroprotective medications including proton pump inhibitors or histamine-2 receptor antagonists [46]. Overall this study, even if it was open label and not rigorously controlled, shows that flavocoxid possesses a significant efficacy in the management of OA with a good safety profile [46].

## 8. Conclusions

**Figure 4:**
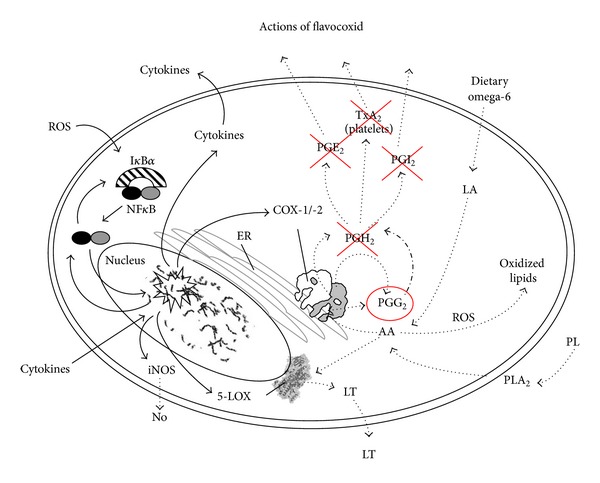
Mode of action of flavocoxid on cellular pathways.

Steroidal anti-inflammatory drugs and nonsteroidal anti-inflammatory drugs are effective in the management of the acute inflammatory reaction, but they do not influence successfully chronic inflammatory states and possess severe adverse effects. Flavocoxid is a mixture of the flavonoids molecules catechin, from* Acacia catechu*, and baicalin, extracted from* Scutellaria baicalensis*, concentrated to greater than 90% purity. Flavocoxid hind PLA_2_ causes a balanced inhibition of the COX-1 and COX-2 peroxidase moieties and decreases the generation of LTBs from 5-LOX, avoiding the deleterious accumulation of these lipid mediators caused by the NSAIDs-induced 5-LOX shunt. It has also a strong antioxidant activity, and through NF-*κ*B activity inhibition blocks the amplifying loop of the inflammatory response and acts at the level of gene and protein expression (Figure 4). It exerts beneficial effects in several experimental models of inflammation.* In vitro* toxicity testing and acute and subchronic toxicity animal studies indicate for flavocoxid an optimal preclinical safety profile. Finally, clinical trials and a postmarketing study show that flavocoxid has a significant efficacy in management of OA and a good overall and GI tolerability. Flavocoxid may therefore provide a potential therapeutic approach to the treatment of acute and chronic inflammatory conditions.

## References

[1] Bengmark S (2004). Acute and “chronic” phase reaction—A mother of disease. *Clinical Nutrition*.

[2] Basu S (2010). Bioactive eicosanoids: role of prostaglandin F_2α_ and F_2_-isoprostanes in inflammation and oxidative stress related pathology. *Molecules and Cells*.

[3] Lawrence T, Gilroy DW (2007). Chronic inflammation: a failure of resolution?. *International Journal of Experimental Pathology*.

[4] Middleton E, Kandaswami C, Theoharides TC (2000). The effects of plant flavonoids on mammalian cells: implications for inflammation, heart disease, and cancer. *Pharmacological Reviews*.

[5] Ray D, Sharatchandra K, Thokchom IS (2006). Antipyretic, antidiarrhoeal, hypoglycaemic and hepatoprotective activities of ethyl acetate extract of *Acacia catechu* Willd. in albino rats. *Indian Journal of Pharmacology*.

[6] Sawangjaroen N, Sawangjaroen K (2005). The effects of extracts from anti-diarrheic Thai medicinal plants on the *in vitro* growth of the intestinal protozoa parasite: blastocystis hominis. *Journal of Ethnopharmacology*.

[7] Tang L, Wei W, Wang X (2007). Effects and mechanisms of catechin for adjuvant arthritis in rats. *Advances in Therapy*.

[8] Chou TC, Chang LP, Li CY, Wong CS, Yang SP (2003). The antiinflammatory and analgesic effects of baicalin in carrageenan-evoked thermal hyperalgesia. *Anesthesia and Analgesia*.

[9] Chung C-P, Park J-B, Bae K-H (1995). Pharmacological effects of methanolic extract from the root of *Scutellaria baicalensis* and its flavonoids on human gingival fibroblast. *Planta Medica*.

[10] Kim HP, Son KH, Chang HW, Kang SS (2004). Anti-inflammatory plant flavonoids and cellular action mechanisms. *Journal of Pharmacological Sciences*.

[11] Cheng P, Lee Y, Wu Y, Chang T, Jin J, Yen M (2007). Protective effect of baicalein against endotoxic shock in rats in vivo and in vitro. *Biochemical Pharmacology*.

[12] Li BQ, Fu T, Gong W-H (2000). The flavonoid baicalin exhibits anti-inflammatory activity by binding to chemokines. *Immunopharmacology*.

[13] Li BQ, Fu T, Dongyan Y, Mikovits JA, Ruscetti FW, Wang JM (2000). Flavonoid baicalin inhibits HIV-1 infection at the level of viral entry. *Biochemical and Biophysical Research Communications*.

[14] Li B, Fu T, Yan Y, Baylor NW, Ruscetti FW, Kung H (1993). Inhibition of HIV infection by Baicalin-A flavonoid compound purified from Chinese herbal medicine. *Cellular and Molecular Biology Research*.

[15] Nagai T, Moriguchi R, Suzuki Y, Tomimori T, Yamada H (1995). Mode of action of the anti-influenza virus activity of plant flavonoid, 5,7,4′-trihydroxy-8-methoxyflavone, from the roots of Scutellaria baicalensis. *Antiviral Research*.

[16] Chang W, Shao Z, Yin J (2007). Comparative effects of flavonoids on oxidant scavenging and ischemia-reperfusion injury in cardiomyocytes. *European Journal of Pharmacology*.

[17] Shao Z, Li C, Vanden Hoek TL (1999). Extract from Scutellaria baicalensis Georgi attenuates oxidant stress in cardiomyocytes. *Journal of Molecular and Cellular Cardiology*.

[18] Shao Z, Vanden Hoek TL, Qin Y (2002). Baicalein attenuates oxidant stress in cardiomyocytes. *American Journal of Physiology - Heart and Circulatory Physiology*.

[19] Zhang DY, Wu J, Ye F (2003). Inhibition of cancer cell proliferation and prostaglandin E2 synthesis by Scutellaria baicalensis. *Cancer Research*.

[20] Kumagai T, Müller CI, Desmond JC, Imai Y, Heber D, Koeffler HP (2007). *Scutellaria baicalensis*, a herbal medicine: anti-proliferative and apoptotic activity against acute lymphocytic leukemia, lymphoma and myeloma cell lines. *Leukemia Research*.

[21] Li BQ, Fu T, Gong W (2000). The flavonoid baicalin exhibits anti-inflammatory activity by binding to chemokines. *Immunopharmacology*.

[22] Bitto A, Minutoli L, David A (2012). Flavocoxid, a dual inhibitor of COX-2 and 5-LOX of natural origin, attenuates the inflammatory response and protects mice from sepsis. *Critical Care*.

[23] Burnett BP, Jia Q, Zhao Y, Levy RM, Chen S (2007). A medicinal extract of *Scutellaria baicalensis* and *Acacia catechu* acts as a dual inhibitor of cyclooxygenase and 5-lipoxygenase to reduce inflammation. *Journal of Medicinal Food*.

[24] Burnett BP, Bitto A, Altavilla D, Squadrito F, Levy RM, Pillai L (2011). Flavocoxid inhibits phospholipase A2, peroxidase moieties of the cyclooxygenases (COX), and 5-lipoxygenase, modifies COX-2 gene expression, and acts as an antioxidant. *Mediators of Inflammation*.

[25] Vardeny O, Solomon SD (2008). Cyclooxygenase-2 Inhibitors, Nonsteroidal Anti-inflammatory Drugs, and Cardiovascular Risk. *Cardiology Clinics*.

[26] Martel-Pelletier J, Lajeunesse D, Reboul P, Pelletier J (2003). Therapeutic role of dual inhibitors of 5-LOX and COX, selective and non-selective non-steroidal anti-inflammatory drugs. *Annals of the Rheumatic Diseases*.

[27] Mao JT, Tsu I, Dubinett SM (2004). Modulation of pulmonary leukotriene B4 production by cyclooxygenase-2 inhibitors and lipopolysaccharide. *Clinical Cancer Research*.

[28] Rainsford KD (1993). Leukotrienes in the pathogenesis of NSAID-induced gastric and intestinal mucosal damage. *Agents and Actions*.

[29] Altavilla D, Squadrito F, Bitto A (2009). Flavocoxid, a dual inhibitor of cyclooxygenase and 5-lipoxygenase, blunts pro-inflammatory phenotype activation in endotoxin-stimulated macrophages. *British Journal of Pharmacology*.

[30] Cunha FQ, Poole S, Lorenzetti BB, Ferreira SH (1992). The pivotal role of tumour necrosis factor α in the development of inflammatory hyperalgesia. *British Journal of Pharmacology*.

[31] Ghosh S, May MJ, Kopp EB (1998). NF-*κ*B and rel proteins: evolutionarily conserved mediators of immune responses. *Annual Review of Immunology*.

[32] Baldwin AS (2001). Series introduction: the transcription factor NF-kappaB and human disease. *The Journal of Clinical Investigation*.

[33] Kopp EB, Ghosh S (1995). NF-*κ*B and rel proteins in innate immunity. *Advances in Immunology*.

[34] Braganza JM, Lee SH, McCloy RF, McMahon MJ (2011). Chronic pancreatitis. *The Lancet*.

[35] Thrower EC, Gorelick FS, Husain SZ (2010). Molecular and cellular mechanisms of pancreatic injury. *Current Opinion in Gastroenterology*.

[36] Polito F, Bitto A, Irrera N (2010). Flavocoxid, a dual inhibitor of cyclooxygenase-2 and 5-lipoxygenase, reduces pancreatic damage in an experimental model of acute pancreatitis. *The British Journal of Pharmacology*.

[37] Messina S, Bitto A, Aguennouz M (2009). Flavocoxid counteracts muscle necrosis and improves functional properties in mdx mice: a comparison study with methylprednisolone. *Experimental Neurology*.

[38] Iannitti T, Capone S, Feder D, Palmieri B (2010). Clinical use of immunosuppressants in duchenne muscular dystrophy. *Journal of Clinical Neuromuscular Disease*.

[39] Messina S, Altavilla D, Aguennouz M (2006). Lipid peroxidation inhibition blunts nuclear factor-kappaB activation, reduces skeletal muscle degeneration, and enhances muscle function in mdx mice. *The American Journal of Pathology*.

[40] Altavilla D, Minutoli L, Polito F (2012). Effects of flavocoxid, a dual inhibitor of COX and 5-lipoxygenase enzymes, on benign prostatic hyperplasia. *British Journal of Pharmacology*.

[41] Burnett BP, Silva S, Mesches MH, Wilson S, Jia Q (2007). Safety evaluation of a combination, defined extract of *Scutellaria baicalensis* and *Acacia catechu*. *Journal of Food Biochemistry*.

[42] Yimam M, Zhao Y, Ma W, Jia Q, Do S, Shin J (2010). 90-Day oral toxicity study of UP446, a combination of defined extracts of Scutellaria baicalensis and Acacia catechu, in rats. *Food and Chemical Toxicology*.

[43] Levy R, Khokhlov A, Kopenkin S (2010). Efficacy and safety of flavocoxid compared with naproxen in subjects with osteoarthritis of the knee—a subset analysis. *Advances in Therapy*.

[44] Levy RM, Khokhlov A, Kopenkin S (2010). Efficacy and safety of flavocoxid, a novel therapeutic, compared with naproxen: a randomized multicenter controlled trial in subjects with osteoarthritis of the knee. *Advances in Therapy*.

[45] Pillai L, Levy RM, Yimam M, Zhao Y, Jia Q, Burnett BP (2010). Flavocoxid, an anti-inflammatory agent of botanical origin, does not affect coagulation or interact with anticoagulation therapies. *Advances in Therapy*.

[46] Pillai L, Burnett BP, Levy RM (2010). GOAL: Multicenter, open-label, post-marketing study of flavocoxid, a novel dual pathway inhibitor anti-inflammatory agent of botanical origin. *Current Medical Research and Opinion*.

